# Synthesis, the Reversible Isostructural Phase Transition, and the Dielectric Properties of a Functional Material Based on an Aminobenzimidazole–Iron Thiocyanate Complex

**DOI:** 10.3390/ijms25169064

**Published:** 2024-08-21

**Authors:** Yang Liu, Adila Abuduheni, Fang Yang, Hongzhi Hu, Zunqi Liu

**Affiliations:** 1 Chemistry and Chemical Engineering College, Xinjiang Agricultural University, Urumqi 830052, China; ly2021@xjau.edu.cn (Y.L.); 17799751675@163.com (A.A.); 15739563025@163.com (F.Y.); huhongzhi305@163.com (H.H.); 2 Xinjiang Sub-Center National Engineering Research Center of Novel Equipment for Polymer Processing, Urumqi 830052, China; 3 Xinjiang Key Laboratory of Agricultural Chemistry and Biomaterials, Urumqi 830052, China

**Keywords:** iron thiocyanato complexes, 2-aminobenzimidazole, two-dimensional hydrogen-bonded structures, reversible isostructural phase transitions, dielectric properties

## Abstract

By introducing disordered molecules into a crystal structure, the motion of the disordered molecules easily induces the formation of multidimensional frameworks in functional crystal materials, allowing for structural phase transitions and the realization of various dielectric properties within a certain temperature range. Here, we prepared a novel ionic complex [C_7_H_8_N_3_]_3_[Fe(NCS)_6_]**·**H_2_O (**1**) between 2-aminobenzimidazole and ferric isothiocyanate from ferric chloride hexahydrate, ammonium thiocyanate, and 2-aminobenzimidazole using the evaporation of the solvent method. The main components, the single-crystal structure, and the thermal and dielectric properties of the complex were characterized using infrared spectroscopy, elemental analysis, single-crystal X-ray diffraction, powder XRD, thermogravimetric analysis, differential scanning calorimetry, variable-temperature and variable-frequency dielectric constant tests, etc. The analysis results indicated that compound **1** belongs to the *P*2_1_/n space group. Within the crystal structure, the [Fe(NCS)_6_]^3−^ anion formed a two-dimensional hydrogen-bonded network with the organic cation through S**···**S interactions and hydrogen bonding. The disorder–order motion of the anions and cations within the crystal and the deformation of the crystal frameworks lead to a significant reversible isostructural phase transition and multiaxial dielectric anomalies of compound **1** at approximately 240 K.

## 1. Introduction

Disorder–order rotations of cations and anions within a molecular structure and the pendulum motion, reorientation, and rotation of the molecule tend to cause phase transitions and physical property transformations in organic–inorganic hybrid functional materials [1,2,3,4,5]. Organic nitrogen-containing heterocyclic compounds, such as imidazoles and pyridines, have a certain polarity and planar structural rigidity and are prone to motions including dynamic molecular rotations in response to external stimuli [6,7,8,9,10]. Introducing these compounds into the structure of functional materials can be an effective strategy to induce material phase transitions [11,12,13,14,15,16,17]. As a new type of pseudohalogen compound, thiocyanate ligands facilitate the formation of bridged coordination structures in the crystal space. Therefore, the thiocyanate anion forms inorganic complexes with transition metal ions such as cobalt, iron, and manganese through coordination bonds, which can be used to construct an anionic framework with a larger space compared to those formed from monoatomic halogens. This creates the potential for increased dynamic movement of organic cations within a certain structure [18,19,20,21,22,23,24,25]. Hence, thiocyanate plays an important role in the phase transition and induced physical properties of hybrid organic–inorganic materials [26,27,28,29,30,31]. One of the current strategies for designing the structure of hybrid organic–inorganic functional phase change materials (PCMs) is to construct a molecular motion system that is capable of order–disorder phase transitions, rotation, and directional motion [32,33,34,35]. According to previous research, the order–disorder phase transition is currently the most used scheme for designing PCMs, where the random movement of organic cations and inorganic anions is the main trigger for the transition. Such compounds are small, highly flexible, and can undergo order–disorder transitions as the temperature changes from low temperature (LT) to room temperature (RT), resulting in structural phase transitions [36,37,38,39,40]. In summary, the key element in designing hybrid organic–inorganic PCMs is to find available flexible organic cations and moderately sized inorganic anions. With a specific structural design, order–disorder movements and structural phase transitions can easily occur, endowing the resulting materials with excellent physical properties [41,42,43,44]. Therefore, to meet the requirements of modern smart-material development, research on organic–inorganic crystalline PCMs needs to advance towards reversible phase transitions and versatile performance [45,46,47,48]. In 2019, Fu et al. [49] synthesized a new type of material, [(CH_3_)_3_N(CH_2_)_2_Cl]_2_[Mn(SCN)_4_(H_2_O)_2_], with switchable properties that respond to external stimuli. The material exhibited a high-temperature reversible phase transition and second harmonic generation (SHG) effect at 371 K; therefore, it could potentially be used as an SHG dielectric switch. This research provided a starting point for the development of dual dielectric switching and SHG functional materials. In 2022, Huang et al. [50] reported a new organic–inorganic hybrid compound [(CH_3_)_3_NH)_4_[Fe(SCN)_6_]Cl. This material underwent a structural phase transition at a temperature of ≈195 K, accompanied by the thermal hysteresis of dielectric bistability as well as anisotropic dielectric relaxation along the crystallographic *a*, *b*, and *c* axes. Therefore, it was considered a potential relaxor-type dielectric material. As this study systematically analyzed the relationship between the dynamic structure of a molecular-based PCM and its dielectric properties, it proposed an alternative path for the development of functional materials. 2-Aminobenzimidazole, an organic cyclic molecule containing five- and six-membered rings, has clear polarity in a specific direction. The molecule tends to exhibit spatial rotation or pendulum motion in response to external stimuli [51]. The introduction of metal thiocyanate complexes with a certain molecular length can easily generate novel functional materials. Based on this knowledge, we synthesized a novel aminobenzimidazole–iron thiocyanate coordination compound, [C_7_H_8_N_3_]_3_[Fe(NCS)_6_]**·**H_2_O (**1**), via natural evaporation from raw materials including ferric chloride hexahydrate, ammonium thiocyanate, and 2-aminobenzimidazole. Water and acetonitrile were used as solvents, and hydrochloric acid was used as a protonating agent. The main components, the single-crystal structure, and the thermal and dielectric properties of the compound were characterized using infrared (IR) spectroscopy, elemental analysis, single-crystal X-ray diffraction (XRD), powder XRD, thermogravimetric analysis (TGA), differential scanning calorimetry (DSC), variable-temperature and variable-frequency dielectric constant tests, etc. The results indicated that the compound was a novel isostructural phase-transition functional material with dielectric anisotropy.

## 2. Results and Discussion

### 2.1. IR Spectroscopy

**Figure 1:**
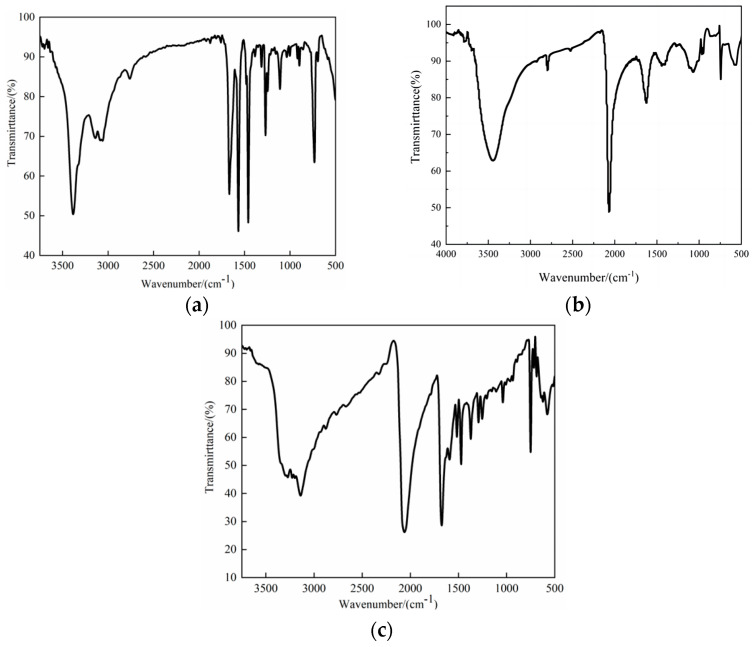
This figure shows the following: (**a**) the infrared spectrum of 2-aminobenzimidazole; (**b**) the infrared spectrum of isothiocyanates; and (**c**) the infrared spectrum of compound **1**.

Infrared spectroscopy can be used to initially confirm the presence of active components in a compound. An appropriate number of clear, regularly shaped compound **1** crystals are mixed with dry potassium bromide in a ratio of 1:100 and ground in a mortar. The fine mixture was pressed into pellets and analyzed in the wave number range of 4000~400 cm^−1^. The infrared spectra of 2-aminobenzimidazole, isothiocyanate raw materials, and compound **1** are shown in Figure 1a–c, respectively. The spectra of the raw material peaked at 742 cm^−1^ and 1108 cm^−1^, corresponding to the out-of-plane and in-plane flexural vibrations of the C-H bond on the aryl ring, respectively. The peaks at 1386, 1453, 1568, and 1662 cm^−1^ are caused by the backbone vibration of the aryl ring, while the peaks at 1269 cm^−1^ and 3396 cm^−1^ are related to the stretching of the C-N bond in the ring and the amino substituents on the ring, respectively. As shown in Figure 1b, in addition to the characteristic peak of the raw material, the -N=C=S tensile vibration peak of ammonium thiocyanate also appeared at 2061 cm^−1^. In summary, compound **1** contains organic molecules and active ingredients such as thiocyanates.

### 2.2. Powder XRD Analysis

**Figure 2:**
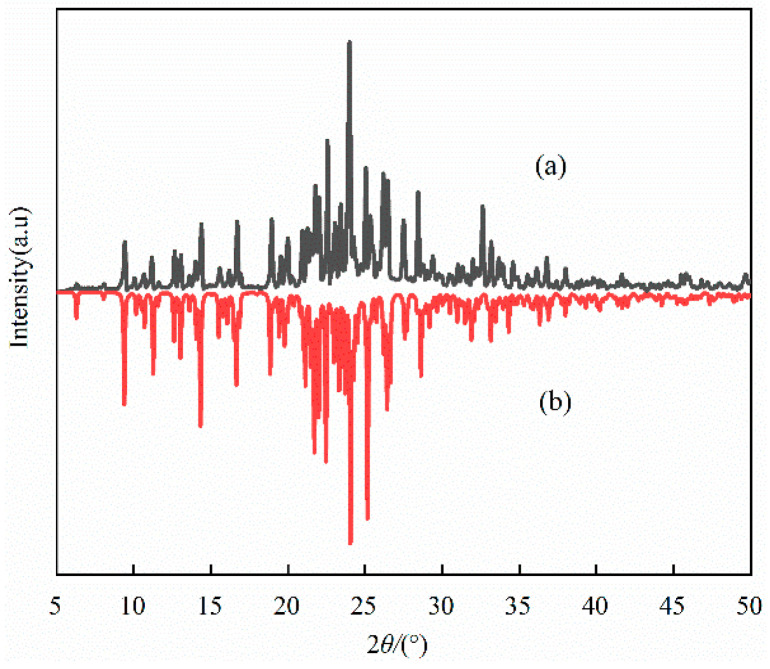
XRD analysis of compound **1**. (**a**) the actual value and (**b**) the simulated value.

Compound **1** (30 mg) is ground into a powder. Powder XRD analysis was performed at RT in the 2*θ* range of 5–50°, and the resulting diffraction pattern is shown in Figure 2a. The theoretical diffraction pattern of compound **1** is shown in Figure 2b, and the single crystal structure data are simulated by Mercury 3.3 software. The experimental XRD pattern shows that compound **1** matches the theoretical diffraction pattern in terms of the intensity and shape of the diffraction peak. This proves that compound **1** has high purity and phase singularity, which supports the reliability of subsequent structural and property tests.

### 2.3. Crystal Structure Analysis

**Figure 3:**
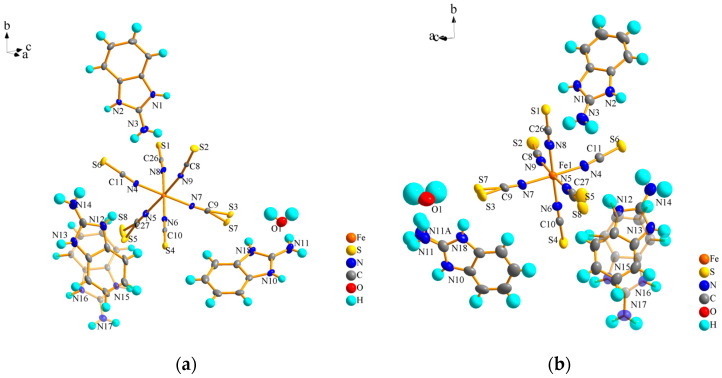
Asymmetric structural units of compound **1** at (**a**) 100 K and (**b**) 293 K.

We selected the right size, a smooth surface, and an unmarked single-crystal sample. Diffraction data for these crystals were acquired at an LT (100 K) and RT (293 K) using a single-crystal X-ray diffractometer equipped with a graphite monochromator that produces mo-K-α radiation (λ = 0.071073 nm) as the diffraction source. The CCDC label is 2333013 at a low temperature and 2333014 at room temperature. Table S1 shows partial crystal diffraction data of compound 1. At low temperatures, compound 1 has the formula [(C7H8N3)]3 [Fe(NCS)6]**·**H2O and belongs to the P21/n space group in the monoclinic system with 100 K crystal cell parameters of *a* = 9.2014(4) Å, *b* = 18.1152(10) Å, *c* = 21.7410 (10) Å, *α* = 90°, *β* = 93.151(5)°, *γ* = 90°, and *V* = 2081.2(2) Å^^3^. The results showed that with the increase in temperature, the cell parameters *a*, *b*, and *c* increased by 0.89%, 0.64%, and 1.19%; *β* decreased by 0.39%, and V increased by 2.69%. This suggests that compound 1 undergoes a potential isostructural phase transition with temperature change. Figure 3a,b show the smallest structural unit of compound **1**, with the thermal ellipsoid set at the 50% probability level at 100 K and 293 K, respectively. The results show that at different temperatures, the crystal cell contains a hexagonal iron thiocyanate complex [Fe(NCS)_6_] anion, three protonated 2-aminobenzimidazole cations and a free water molecule, one of which exists in a disordered state at an LT and RT. Bond lengths and bond angles of cations and anions are shown in Supplementary Table S2.

**Figure 4:**
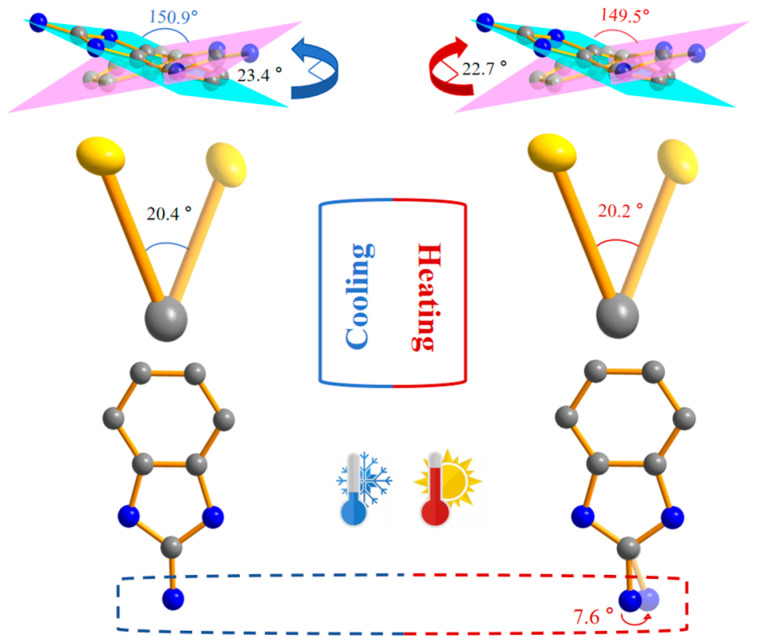
Structures of the order–disorder unit cells of compound **1** at 100 K and 293 K.

Figure 4 shows that the 2-aminobenzimidazole cation randomly rotated at an LT and RT. The occupancy rates of C19~C25/N12~N14 and C19B~C25B/N15~N17 were 0.617 and 0.383, respectively, at an LT, and 0.611 and 0.389, respectively, at RT. The results show that as the temperature increased, the occupancy rates of the disordered atoms in the 2-aminobenzimidazole gradually approached each other, indicating clear disorder-to-disorder transitions of the molecule within the spatial structure. Analysis of the internal structure of the organic cation exhibiting random motions showed that the angle between the two planes created by the disordered 2-aminobenzimidazole was 150.9° at an LT, which changed to 149.5° at RT. This change indicates the folding and oscillating motions of the dihedral angle within the spatial structure, reminiscent of the continuous up and down strokes of butterfly wings. Therefore, it can be speculated that the inorganic anions constructed a large crystal space, making the random motion of 2-aminobenzimidazole cations possible (Supplementary Figure S1).

The inorganic [Fe(NCS)_6_] anion in compound **1** has two S atoms that undergo order–disorder transitions during the change from an LT to RT (Figure 4). Each S atom that underwent the transition occupied two positions at an LT: the occupancy rates of S7~S8 were both 0.383, while those of S3~S5 were both 0.617. At RT, the S7~S8 occupancy rates both changed to 0.389, while those of S3~S5 both changed to 0.611. The occupancy rates gradually approached each other as the temperature rose. The angles formed by the disordered S atoms were 20.4° (LT) and 20.2° (RT), which decreased as the temperature rose. In contrast to the disordered 2-aminobenzimidazole cation, the amino group in another protonated 2-aminobenzimidazole was completely ordered at an LT. However, the N atom in this amino group underwent an order–disorder transition as the temperature rose, with the occupancy rate of N11 changing from 1 at an LT to 0.67, while the occupancy rate of N11A increased to 0.33. The angle between the two sites of the atom occupancy was 7.6°, and the atom oscillated, swinging back and forth in the microstructural space (Figure 4).

**Figure 5:**
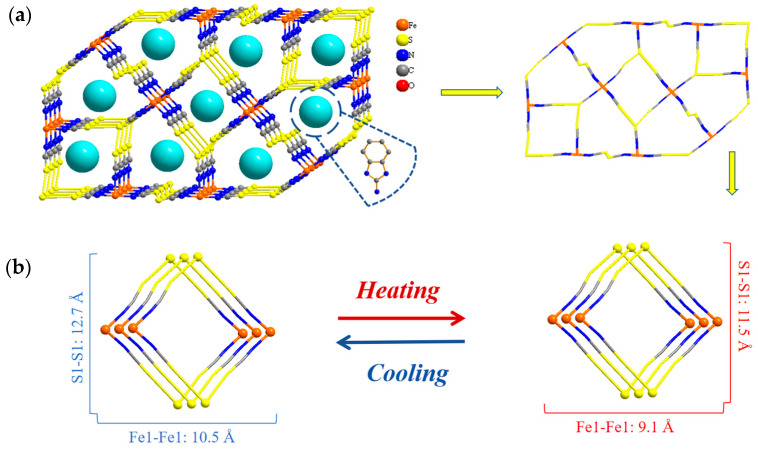
(**a**) The porous mesh structure of compound **1** in the *bc* plane; (**b**) changes in the pore structure.

The structural units of the neighboring inorganic anions extended continuously along the *b* and *c* axes through S**···**S interactions within the structure of compound **1**. These units were stacked in the *bc* plane, constructing a two-dimensional (2D) mesh-channel structure, as shown in Figure 5a. With the change in temperature, the dynamic stretching of the spacing between the S**···**S interactions led to the deformation of the tetragonal framework, where the Fe and S atoms were the vertices in the 2D porous mesh structure (Figure 5b). Specifically, the length of the framework changed from 10.499 Å at an LT to 9.141 Å at RT, and the width changed from 12.679 Å at an LT to 11.523 Å at RT. This indicated that the distance between the iron and sulfur ions in the center of the iron thiocyanate anion in the planar porous structure decreased as the temperature increased. The tetragonal framework also deformed and changed in size to some extent with the temperature change. These findings indicate that the 2D mesh structure underwent internal stretching vibrations as the temperature varied.

**Figure 6:**
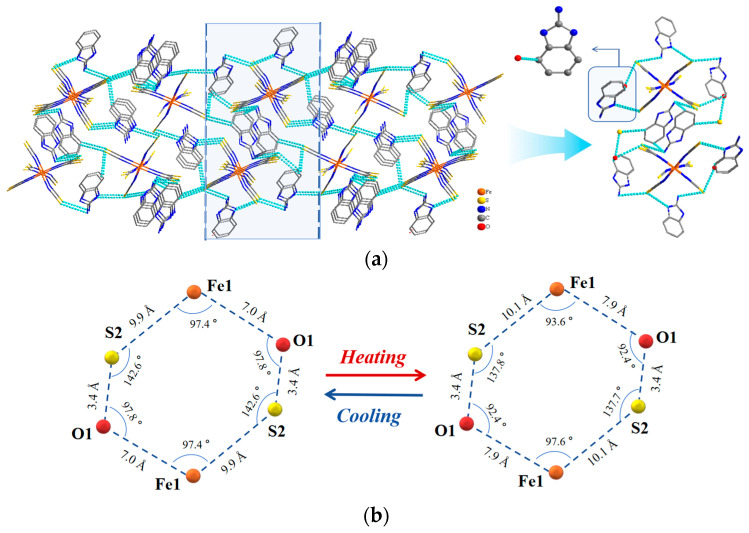
(**a**) A 2D hydrogen-bonded stacked structure of compound **1** in the *bc* plane; (**b**) change in the spatial structure.

A diagram of the 2D hydrogen-bonded stacked structure of the iron thiocyanate anion and protonated 2-aminobenzimidazole in the *bc* plane of compound **1** is shown in Figure 6a. The diagram illustrates the different connections among the transverse and longitudinal chains in the mesh structure. The structural units in the longitudinal chain were connected by N–H**···**S and O–H**···**S hydrogen bonds and extended infinitely along the *b*-axis to form a 2D hydrogen-bonded stacked structure in a “heart” shape. The average length of the N–H**···**S and O–H**···**S hydrogen bonds was 3.409 Å, and the average bond angle was 140.7° at an LT (Table 1). At RT, the average bond length and angle were 3.456 Å and 140.2°, respectively, and the bond length increased by 1.38% after the temperature change. The transverse chains were connected by the N–H**···**S hydrogen bonds and extended infinitely along the direction of the *c*-axis to form a 2D hydrogen-bonded stacked structure. The average length of the N-H**···**S hydrogen bonds was 3.448 Å, with an average hydrogen bond angle of 147.9° at an LT (Table 1). At RT, the average bond length was 3.516 Å, and the average bond angle was 146.9°; after the temperature change, the bond length increased by 1.45%. These results showed that the longitudinal and transverse chain lengths increased in the *bc* plane. As shown in Figure 6a, this structure formed a stacked mesh layer through hydrogen bonding, with the free water molecules as well as the organic protonated 2-aminobenzimidazole acting as transverse and longitudinal attachment points (Supplementary Figure S2). Figure 6b shows the changes in the spatial structure of compound **1**. Five neighboring atoms (Fe1, S1, and O1) in the same plane were selected from the stacked layer as the vertices to form a pentagonal framework. The lengths of the pentagon sides increased, while the angles decreased as the temperature rose. Such phenomena indicated that the increase in temperature led to the deformation of the structural pentagonal framework constructed with the iron thiocyanate complexes. Moreover, the bond lengths and angles in compound **1** also changed, which provides favorable conditions for the manifestation of dielectric properties.

### 2.4. Hirshfeld Surface Analysis

**Figure 7:**
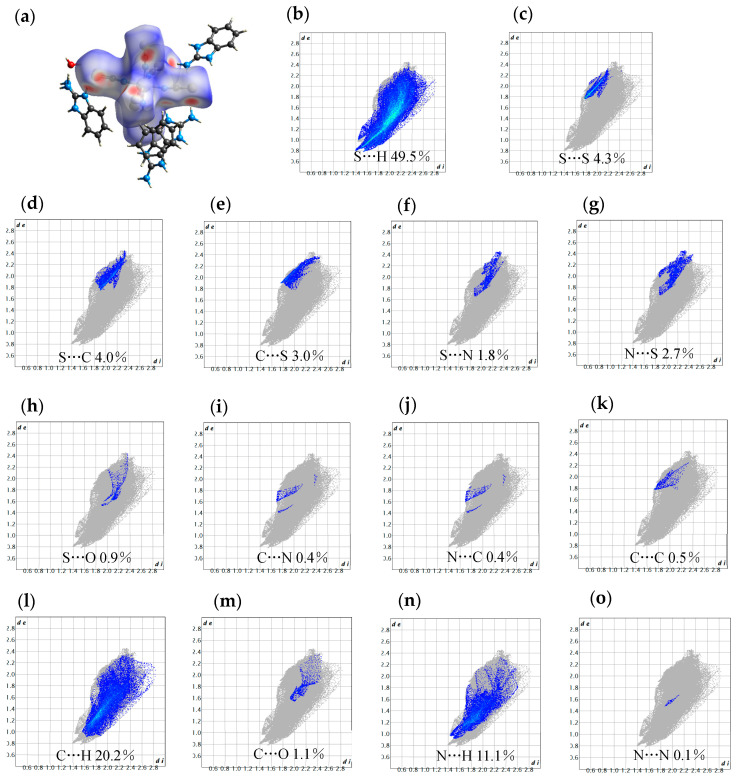
Hirshfeld surface diagram (**a**) of compound **1**, and 2D finger pattern formed with *dnorm* as parameter (**b**–**o**).

In order to further determine the structural differences of the interactions between molecules, the interactions between atoms in the crystal structure are calculated and analyzed. Hirshfeld surface is an important and common means to analyze the interaction between atoms in the crystal structure. In this paper, crystalexplorer software is used for analysis and calculation based on the DFT principle. The atoms of the 2-aminophenimidazole and iron isothiocyanate complex were selected for modeling. Then, the normalized interatomic interaction (*dnorm*) was used as the parameter to image the Hirshfeld surface along the 2D fingerprints, and the general situation of the interaction forces between different atoms was obtained. As shown in Figure 7a, the Hirshfeld surface contains a molecule of ferric isothiocyanide. Each interaction can be quantified by finger pattern, as shown in Figure 7b–o. It can be seen from the finger pattern that the interaction between S**···**H accounts for 49.5% of the entire crystal, and the interaction between S**···**S accounts for 4.3% of the entire crystal. The interaction between S**···** C and C**···** S accounts for 7.0% of the entire crystal, the interaction between S**···**N and N**···**S accounts for 4.5% of the entire crystal, the interaction between S**···**O accounts for 0.9% of the entire crystal, and the interaction between C**···**N and N**···**C accounts for 0.8% of the entire crystal. The interaction between C**···**C, C**···**H, and C**···**O accounts for 21.8% of the entire crystal, and the interaction between N**···**H and N**···**N accounts for 11.2% of the entire crystal. Therefore, the results show that there is a strong hydrogen bond interaction force between the molecules of compound **1**, which is consistent with the results of crystal structure analysis that there is a two-dimensional hydrogen bond network structure in the complex.

### 2.5. UV-Vis Spectral Analysis

**Figure 8:**
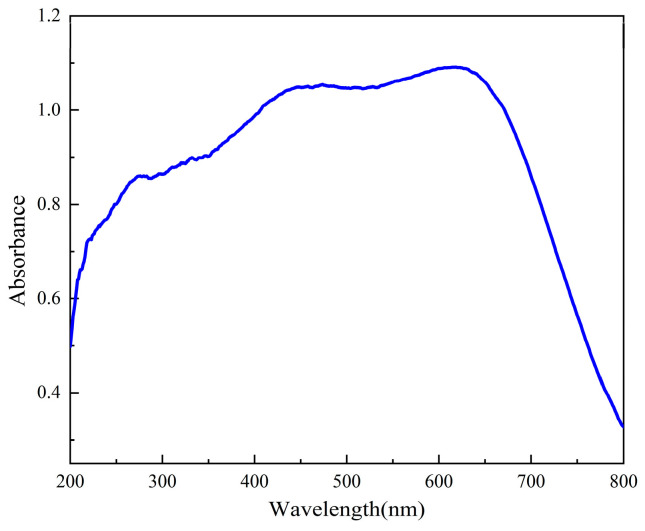
Uv spectrum determination of compound **1**.

In order to further detect its optical properties, the ultraviolet spectrum of compound **1** was tested in the 200–800 nm region. As shown in Figure 8, compound **1** showed different electronic transition behaviors. [C_7_H_8_N_3_]_3_[Fe(NCS)_6_]**·**H_2_O (**1**) showed UV absorption peaks at 280, 350, 450, and 620 nm. The absorption peaks between 250 nm and 280 nm are all caused by the π→π* electron transition of the conjugated structure and benzene ring in [C_7_H_8_N_3_]_3_, and the σ-π* transition of NCS^−^. In the structure, the presence of its amino group interferes with and affects the electronic properties of the molecule, so that it changes the spectral properties, and [C_7_H_8_N_3_]_3_ exhibits an absorption peak at 350 nm, usually associated with aromatic rings and conjugated structures. There are two strong absorption peaks near 450 and 620 nm, both of which are caused by the intermolecular interaction in [Fe(NCS)_6_]. The above strong absorption peaks indicate that compound **1** has high selectivity at specific wavelengths, that is, it can effectively absorb specific wavelengths of light and has good light stability. The results are consistent with the structural changes, and therefore compound **1** has good electron transfer performance.

### 2.6. TGA

**Figure 9:**
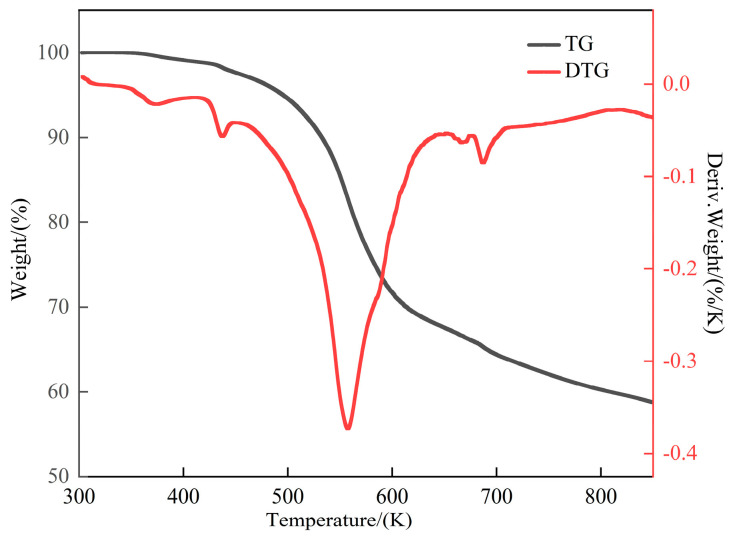
TG and DTG curves of compound **1**.

The thermal stability of the crystals influences the potential applications of the resulting materials. The thermal stability of compound **1** was evaluated using a thermogravimetric analyzer under nitrogen protection within the temperature range of 300~850 K at a heating rate of 10 K min^−1^. The obtained TG and differential TG (DTG) curves are shown in Figure 9. The results show two stages of compound **1** decomposition. The first one at 320~355 K involved a 0.02% weight loss, which corresponds with the loss of one crystalline water molecule (the loss of one water molecule within the crystal results in a theoretical weight loss of 0.02%). The second decomposition stage occurred at 355~800 K, with a weight loss that matches the theoretical weight loss resulting from the decomposition of the three protonated 2-aminobenzimidazole cations in compound **1** (theoretical weight loss of 48.33%). A significant endothermic peak was observed in the DTG curve in this temperature range. The crystals gradually stabilized after reaching 800 K, characterized by a deceleration in the decomposition rate, resulting in a final residue comprising 49.67% of the original weight. From the crystal structure, the residue was speculated to be iron thiocyanate complexes or iron oxides that did not decompose. After analyzing the TG and DTG curves of compound **1**, the results showed that the actual weight-loss rate of the crystal generally matched the theoretical weight-loss rate within the temperature range tested. The chemical components of compound **1** derived from the thermal decomposition curve were generally consistent with the molecular formula of [C_7_H_8_N_3_]_3_[Fe(NCS)_6_]**·**H_2_O, as obtained in the single-crystal XRD analysis.

### 2.7. DSC Analysis

**Figure 10:**
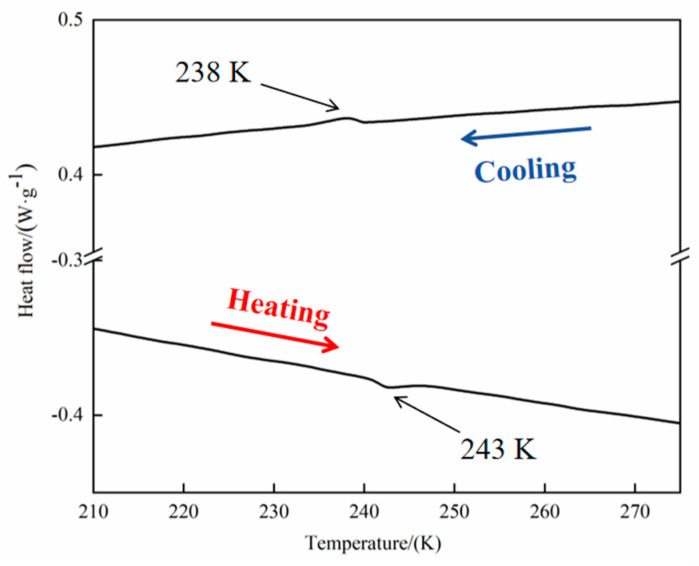
DSC curve of compound **1**.

Thermal phase transitions in crystals can help materials acquire good optical and dielectric properties within a certain temperature range. DSC is a fundamental method for determining the presence or absence of solid structural transitions in molecular crystals induced by thermal stimuli. To further confirm the isostructural phase transition of compound **1**, it was analyzed in the temperature range of 210–280 K under nitrogen with a temperature ramp of 5 K/min; the results are shown in Figure 10. The DSC curve of the cooling–heating cycle showed a significant endothermic peak near T1 = 238 K during the cooling process and a significant exothermic peak near T_2_ = 243 K during the warming process. Therefore, a reversible thermal energy cycle occurs near 240.5 K, with a thermal hysteresis of ≈5 K. The hysteresis phenomenon during the thermal phase transition within the crystal structure implies the existence of an intermediate phase during the degradation of the crystal symmetry. Thus, the phase transition appears to have been completed in a single step, with sharp phase-transition peaks and thermal hysteresis, indicating that compound **1** underwent a first-order phase transition. The entropy change (Δ*S*) was Δ*S*_1_ ≈ 5.53 J mol^−1^ K^−1^ for the phase transition in the reversible process and Δ*S*_2_ = 6.15 J mol^−1^ K^−1^ for the warming process. Based on Δ*S* = *nR ln*N, N_1_ = 1.95 and N_2_ = 2.1 for the cooling and warming processes, respectively. According to the Boltzmann equation, the ratio of probabilities of microscopic states, N, is ≈2. A relatively large N value indicates that disordered–ordered molecular rotations occur in the crystal within a certain temperature range, which induces a distinct isostructural phase transition in the crystalline material. These results validate that compound **1** is a type of reversible disorder–order PCM, which is in complete agreement with the findings of the variable-temperature analysis of the crystalline material structure.

### 2.8. Dielectric Property Tests

**Figure 11:**
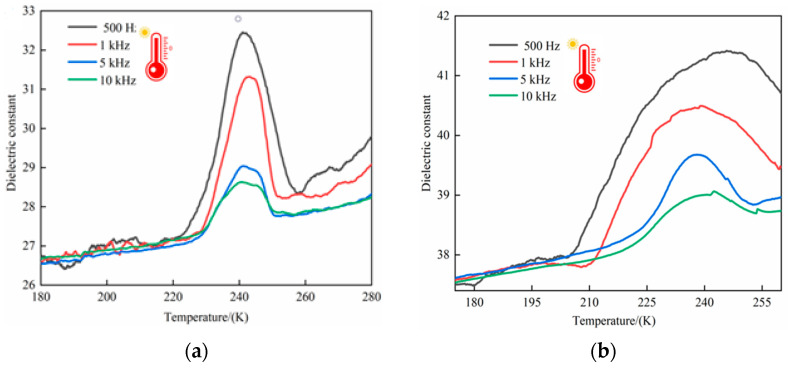

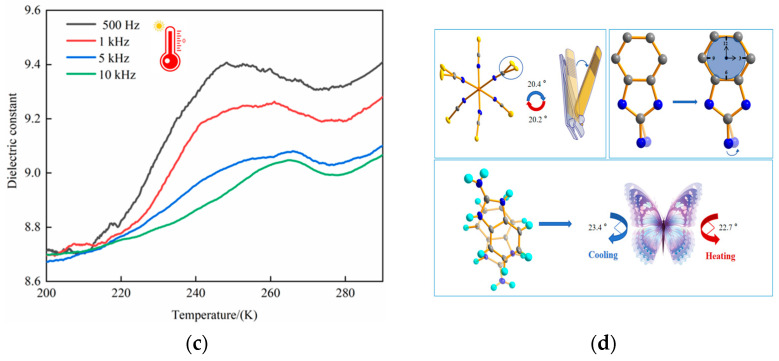
(**a**–**d**) Dielectric constant curves of compound **1** in the *a*, *b*, and *c* axes.

The reversible phase transition of functional materials must be accompanied by anomalies in the dielectric properties. Detecting the changes and anomalies in the dielectric constants of crystalline materials with temperature–frequency variations is important for evaluating the potential application value of the functional materials. Crystals of compound **1** with a regular shape were selected, and the directions of the *a*, *b*, and *c* axes were determined using a single-crystal X-ray diffractometer. The crystals were secured to a special disk of an electrode socket by conductive copper wires and silver glue to construct a capacitor for testing. Variable-temperature and variable-frequency dielectric constant tests were then performed in the temperature range of 180~280 K and frequency range of 500 Hz to 10 KHz. Significant dielectric anomalies were observed in all three axial directions of compound **1** during the warming process, as shown in the dielectric curves in Figure 11a–c. In the *a*-axis direction of compound **1** during the warming process, no changes in the dielectric constant were observed in the low-temperature range of 180~220 K at all frequencies. By contrast, the dielectric constant rapidly increased within the temperature range of 220~260 K, peaking at ≈240 K, and rapidly decreased until the temperature reached 260 K. Afterwards, the dielectric constant increased slowly. In the *b*-axis of compound **1**, the dielectric constant remained the same in the low-temperature range of 180~205 K, increased rapidly once the temperature reached 210 K, peaked at 245 K, and reached a plateau afterwards. In the *c*-axis direction of compound **1**, the changes in the dielectric constant were similar to those of the *a* and *b* axes. Specifically, the dielectric constant remained stable in the temperature range of 180~220 K at all frequencies. As the temperature rose, the dielectric constant increased rapidly, reaching a maximum at ≈250 K, which was accompanied by an intense and wide dielectric anomaly peak. The comprehensive analysis of the changes in dielectric constants along all three axes indicated anisotropy of compound **1**. Moreover, the dielectric constants in different axial directions showed an overall gradual decreasing trend as the frequency increased. This is consistent with the positions of the endothermic peak at 238 K and the exothermic peak at 243 K observed in the DSC phase-transition curve of compound **1**. It is inferred from the thermal energy curves and structure of the crystalline materials that the structural thermal-energy phase transitions and dielectric anomalies in all directions of the material are due to the deformation of the mesh framework induced by the disordered–ordered rotation of the 2-aminobenzimidazole molecules, as well as the changes in hydrogen bond lengths and the angles between the cations and anions comprising the crystal structure.

## 3. Materials and Methods

### 3.1. Reagents and Instruments

Ferric chloride hexahydrate, ammonium thiocyanate, and 2-aminobenzimidazole were purchased from Shanghai Maikelin Biochemical Technology Co., Ltd., Shanghai, China; acetonitrile and hydrochloric acid (36.5 wt%) were purchased from Tianjin Zhiyuan Chemical Reagent Co., Ltd. (Tianjin, China). The reagents used in the experiment were not further processed in the lab.

### 3.2. Synthesis of Compound **1**

**Figure 12:**
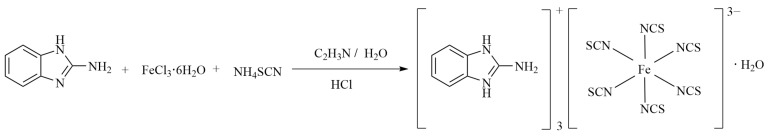
Synthetic route of compound **1**.

Compound **1** was obtained at RT (293 K) using a solvent-evaporation method based on the synthetic route shown in Figure 12. Specifically, 0.20 g (0.74 mmol) of ferric chloride, 0.34 g (4.44 mmol) of ammonium thiocyanate, and 0.19 g (1.48 mmol) of 2-aminobenzimidazole were accurately weighed, resulting in a molar ratio of 1:6:2. The weighed ferric chloride and ammonium thiocyanate were dissolved separately in 10 mL of distilled water and set aside for future use. 2-Aminobenzimidazole was dissolved in 10 mL of acetonitrile, to which 0.5 mL of hydrochloric acid was added dropwise. The mixture was stirred for 10 min on a constant-temperature magnetic stirrer to drive the reaction to completion. Ammonium thiocyanate solution was first added dropwise to a ferric chloride solution, followed by the slow, dropwise addition of a protonated 2-aminobenzimidazole solution. The vessel containing the solution was sealed and transferred to a well-ventilated area for slow evaporation. Black, lumpy crystals were observed at the bottom of the vessel 7 days later. The yield of the synthetic crystal material is 83%. Elemental analysis was performed and revealed a chemical formula of C_27_H_26_FeN_15_OS_6_, with calculated values (%) of C 39.32, H 3.18, and N 25.47, and measured values (%) of C 39.13, H 3.03, and N 25.21.

## 4. Conclusions

This study is based on the design principle that the disordered–ordered motion of organic molecules within the crystal structure and deformation of the framework constructed by anions and cations can lead to structural phase transitions and changes in the dielectric properties of materials. We used ferric chloride hexahydrate and 2-aminobenzimidazole as the raw materials, ammonium thiocyanate as the pseudohalogen linker, hydrochloric acid as the proton donator, and water and acetonitrile as the solvents. Relying on the multi-component solubility of the solvents, crystals of [C_7_H_8_N_3_]_3_[Fe(NCS)_6_]**·**H_2_O (**1**) were obtained for the first time at a temperature of 25 °C through natural evaporation. The results of the structural analysis and property characterization showed that all crystals of compound **1** during the temperature change belonged to the *P*2_1_/n space group in the monoclinic crystal system. The adjacent [Fe(NCS)_6_]^3−^ anions in the structure extended indefinitely in the direction of the *b* and *c* axes through S**···**S interactions to build 2D porous molecular channels with 2-aminobenzimidazole encapsulated inside. Meanwhile, the [Fe(NCS)_6_]^3−^ anions interacted with water and 2-aminobenzimidazole molecules via N-H**···**S and O-H**···**S hydrogen bonds to form a 2D hydrogen-bonded mesh network in the *bc* plane. The DSC thermal energy curve analysis of compound **1** showed a significant endothermic peak at ≈238 K during the cooling process and a significant exothermic peak at ≈243 K during the warming process. The compound exhibited a reversible thermal energy cycling pattern and disordered–ordered molecular rotation in the temperature range of 210~270 K. These results are consistent with the finding that compound **1** showed dielectric anomalies at ≈240 K along the *a*, *b*, and *c* axes of the crystal during the warming process. Reversible isostructural phase transitions and electrical property anomalies in functional materials arise mainly from disordered–ordered molecular motions of 2-aminobenzimidazole cations and [Fe(NCS)_6_]^3−^ anions in the structure, as well as the deformation of hydrogen-bonded frameworks under thermal stimuli. In conclusion, by analyzing the structure and relevant properties of compound **1**, compound **1** was shown to be a reversible isostructural phase-transition functional material with a hydrogen-bonded mesh structure and dielectric anomalies.

## Figures and Tables

**Table 1:** Hydrogen bonding parameters of compound **1** at 100 K and 293 K.

D-H**···**A	*d*(D-A)/Å	$\left|\delta =\bar{x}-\mu \right|$%	∠D-H**···**A/(°)	$\left|\delta =\bar{x}-\mu \right|$%
100 K				
N14-H14**···**S6	3.3	7.1%	164.6	4.7%
N3-H3A**···**S6	3.3	7.1%	173.0	13.3%
C18-H18**···**S3	3.4	2.9%	167.4	7.7%
O1-H1B**···**S3	3.2	17.1%	143.2	16.5%
O1-H1C**···**S5	3.6	22.9%	145.7	14.0%
N3-H3B**···**S2	3.4	2.9%	166.2	6.5%
N2-H2**···**S1	3.4	2.9%	157.8	1.9%
293 K				
N14-H14**···**S6	3.3	8.6%	163.2	11.3%
N3-H3A**···**S6	3.3	8.6%	171.2	19.3%
C18-H18**···**S3	3.4	1.4%	163.7	11.8%
O1-H1B**···**S3	3.2	18.6%	119.8	32.0%
O1-H1C**···**S5	3.6	21.4%	121.6	30.2%
N3-H3B**···**S2	3.4	1.4%	166.9	15.1%
N2-H2**···**S1	3.5	11.4%	156.4	4.6%

## Data Availability

The crystallographic information files (CIFs) have been deposited in the Cambridge Crystallographic Centre (CCDC) database and can be obtained free of charge at https://www.ccdc.cam.ac.uk, accessed on 1 July 2024, (or from the CCDC, 12 Union Road, Cambridge CB2 1EZ, UK; Fax: +44-1223-336033; E-mail: deposit@ccdc.cam.ac.uk) with the following depository number: the CCDC label is 2333013 at a low temperature and 2333014 at room temperature.

## Supplementary Materials

The following supporting information can be downloaded at https://www.mdpi.com/article/10.3390/ijms25169064/s1.
