# Supplementary material for: Synthesis, the Reversible Isostructural Phase Transition, and the Dielectric Properties of a Functional Material Based on an Aminobenzimidazole–Iron Thiocyanate Complex
Source: Int J Mol Sci. 2024 Aug 21;25(16):9064. doi: 10.3390/ijms25169064 (PMC11354588; doi:10.3390/ijms25169064)
Supplement: Supplementary file 1 [file ijms-25-09064-s001.zip › ijms-3111335-supplementary.pdf]

# Synthesis, the reversible isostructural phase transition, and the dielectric properties of a functional material based on an aminobenzimidazole–iron thiocyanate complex

**Table S1 Crystallographic data for compound 1.**

| Temperature | 100 K | 293 K |
|---|---|---|
| Chemical formula | $C_{27}H_{26}FeN_{15}OS_6$ | $C_{27}H_{26}FeN_{15}OS_6$ |
| Formula weight | 824.84 | 824.84 |
| Crystal size ($mm^3$) | 0.15×0.13×0.12 | 0.15×0.13×0.12 |
| Crystal system | monoclinic | monoclinic |
| Space group | $P2_1/n$ | $P2_1/n$ |
| $a$ (Å) | 9.2014(4) | 9.2833(12) |
| $b$ (Å) | 18.1152(10) | 18.231(3) |
| $c$ (Å) | 21.7410(10) | 21.980(3) |
| $\alpha$ ( °) | 90 | 90 |
| $\beta$ ( °) | 93.151(4) | 92.785(12) |
| $\gamma$ ( °) | 90 | 90 |
| $V$ ($Å^3$) | 3618.4(3) | 3715.6(9) |
| $Z$ | 4 | 4 |
| $Dcalc$ ($g{\cdot}cm^{-1}$) | 1.514 | 1.474 |
| $F$(000) | 1692 | 1692 |
| $\mu$ ($mm^{-1}$) | 0.811 | 0.790 |
| Measured $2\theta$ range (º) | 1.000-24.997 | 1.000-24.999 |
| $Rint$ | 0.0401 | 0.0489 |
| $R$ ($I > 2(I)$)[1] | 0.0557 | 0.0758 |
| $\omega R$ (all data)[2] | 0.0973 | 0.1268 |
| GOF | 1.034 | 1.029 |
| CCDC | 2333013 | 2333014 |

[1] $R=\sum(|F_o|-|F_c|)/\sum|F_o|$
[2] $wR=[\sum w(|F_o|^2-|F_c|^2)^2/\sum w(F_o^2)]^{1/2}$

**Table S2 Selected bond lengths (Å) and bond angles(°) of compound 1 at 100 and 293 K**

| chemical bond | Bond length/Å | chemical bond | bond angle/° | chemical bond | bond angle/° |
|---|---|---|---|---|---|
| | | 100 K | | | |
| Fe1-N4 | 2.063(2) | N4-Fe1-N6 | 88.97(9) | C8-N9-Fe1 | 167.6(2) |
| Fe1-N5 | 2.053(2) | N4-Fe1-N8 | 90.22(9) | N9-C8-S2 | 178.5(2) |

| | | | | | |
|---|---|---|---|---|---|
| Fe1-N6 | 2.063(3) | N5-Fe1-N4 | 88.96(9) | N7-C9-S3 | 172.9(3) |
| Fe1-N7 | 2.034(2) | N5-Fe1-N6 | 91.54(10) | N7-C9-S7 | 166.6(3) |
| Fe1-N8 | 2.078(3) | N5-Fe1-N8 | 89.14(10) | N6-C10-S4 | 179.5(3) |
| Fe1-N9 | 2.047(2) | N6-Fe1-N8 | 178.93(9) | N4-C11-S6 | 178.7(3) |
| S1-C26 | 1.629(3) | N7-Fe1-N4 | 178.99(9) | N8-C26-S1 | 178.4(3) |
| S2-C8 | 1.628(3) | N7-Fe1-N5 | 92.04(9) | N5-C27-S5 | 173.2(3) |
| S3-C9 | 1.597(4) | N7-Fe1-N6 | 91.08(10) | N5-C27-S8 | 168.4(4) |
| S4-C10 | 1.632(3) | N7-Fe1-N8 | 89.72(9) | | |
| S5-C27 | 1.642(5) | N7-Fe1-N9 | 90.96(9) | | |
| S6-C11 | 1.632(3) | N9-Fe1-N4 | 88.03(9) | | |
| S7-C9 | 1.705(5) | N9-Fe1-N5 | 176.80(9) | | |
| S8-C27 | 1.661(6) | N9-Fe1-N6 | 89.51(9) | | |
| N4-C11 | 1.158(3) | N9-Fe1-N8 | 89.77(9) | | |
| N5-C27 | 1.152(4) | C11-N4-Fe1 | 168.0(2) | | |
| N6-C10 | 1.161(4) | C27-N5-Fe1 | 171.6(3) | | |
| N7-C9 | 1.156(3) | C10-N6-Fe1 | 169.5(2) | | |
| N8-C26 | 1.164(3) | C9-N7-Fe1 | 173.2(2) | | |
| N9-C8 | 1.160(3) | C26-N8-Fe1 | 165.1(2) | | |
| | | | 293 K | | |
| Fe1-N4 | 2.066(3) | N4-Fe1-N8 | 90.02(12) | C8-N9-Fe1 | 168.6(3) |
| Fe1-N5 | 2.057(3) | N5-Fe1-N4 | 88.76(12) | N9-C8-S2 | 178.2(3) |
| Fe1-N6 | 2.057(3) | N5-Fe1-N6 | 91.02(13) | N7-C9-S3 | 172.7(4) |
| Fe1-N7 | 2.038(3) | N5-Fe1-N8 | 89.08(13) | N7-C9-S7 | 167.0(5) |
| Fe1-N8 | 2.071(3) | N6-Fe1-N4 | 89.08(12) | N6-C10-S4 | 179.4(4) |
| Fe1-N9 | 2.045(3) | N6-Fe1-N8 | 179.10(12) | N4-C11-S6 | 179.0(3) |
| S1-C26 | 1.624(4) | N7-Fe1-N4 | 178.70(13) | N8-C26-S1 | 178.1(3) |
| S2-C8 | 1.618(4) | N7-Fe1-N5 | 92.48(12) | N5-C27-S5 | 171.6(5) |
| S3-C9 | 1.606(8) | N7-Fe1-N6 | 91.30(12) | N5-C27-S8 | 164.1(5) |
| S4-C10 | 1.632(4) | N7-Fe1-N8 | 89.59(12) | | |
| S5-C27 | 1.660(6) | N7-Fe1-N9 | 90.60(12) | | |
| S6-C11 | 1.632(4) | N9-Fe1-N4 | 88.16(12) | | |
| S7-C9 | 1.667(11) | N9-Fe1-N5 | 176.75(12) | | |
| S8-C27 | 1.641(8) | N9-Fe1-N6 | 89.96(12) | | |
| N4-C11 | 1.146(4) | N9-Fe1-N8 | 89.90(12) | | |
| N5-C27 | 1.133(4) | C11-N4-Fe1 | 167.3(3) | | |
| N6-C10 | 1.144(4) | C27-N5-Fe1 | 172.9(4) | | |
| N7-C9 | 1.148(4) | C10-N6-Fe1 | 170.2(3) | | |
| N8-C26 | 1.148(4) | C9-N7-Fe1 | 172.5(3) | | |
| N9-C8 | 1.143(4) | C26-N8-Fe1 | 165.2(3) | | |

This is the hydrogen bond length bond angle table of compound **1**. The results show that **1** is mainly composed of N—H···S、C—H···S and O—H···S. The change of temperature changes the bond length and bond Angle of compound 1 respectively. It shows that the physical and chemical

properties of **1** will change.

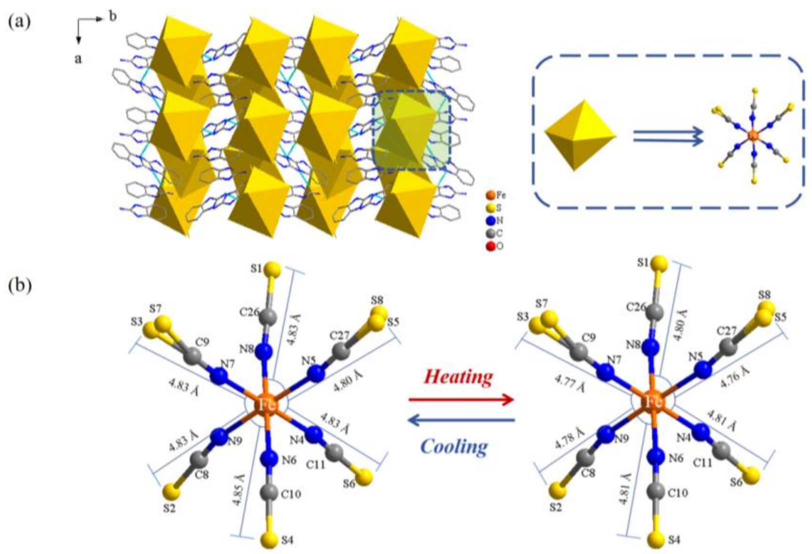


Fig S1 Total arrangement of compound **1** in the *ab* plane (a), anionic structure of inorganic metal-complex (b)

The central ion $Fe^{3+}$ and six $SCN^-$ show a regular octahedral configuration in space. Some of the S atoms (S3, S5, S7 and S8) are disordered, and these S atoms have two sites, and the site occupancy ratio is 0.617/0.383 at low temperature, and the occupancy ratio becomes 0.611/0.389 with the increase of temperature. The increase of temperature causes distortion of metal skeleton $[Fe(NCS)_6]^{3-}$hexahedron structure to a certain extent, which can provide favorable conditions for the disorder and rotation of organic cations in space.

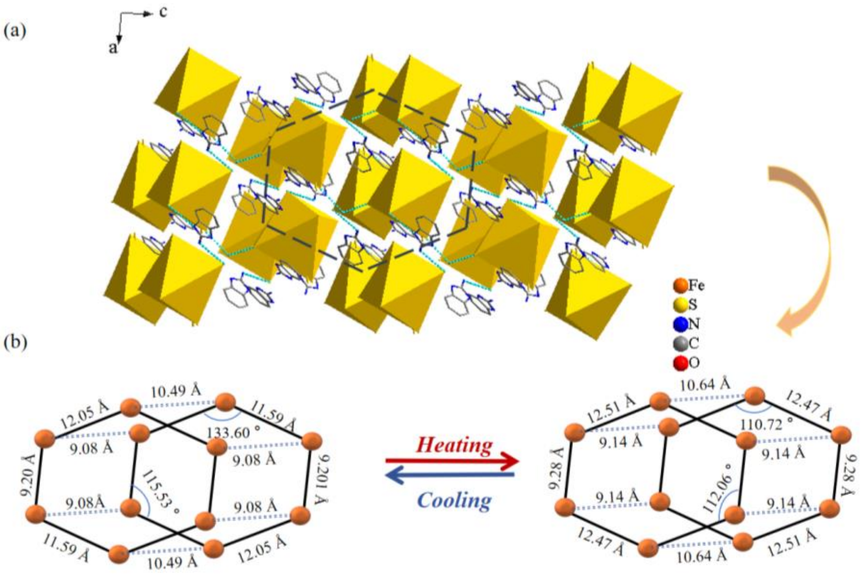


Fig S2 Hydrogen bond packing of compound **1** along the *ac* plane (a), spatial structure diagram (b)

In thi[illegible] are selected to [illegible]sed ring structure in this plane. As the temperature increases, the bond length between the side lengths Fe1-Fe1 of the hexagon increases and the bond Angle is smaller. The results show that the temperature increase results in the deformation of the hexagonal structure frame composed of iron thiocyanate complex.
